# Supplementary material for: Prognostic value of lymphocyte-to-monocyte ratio in patients with endometrial cancer: an updated systematic review and meta-analysis
Source: PeerJ. 2025 Jun 23;13:e19345. doi: 10.7717/peerj.19345 (PMC12199738; doi:10.7717/peerj.19345)
Supplement: Supplemental Information 2 [file peerj-13-19345-s002.docx]

## Systematic Review and/or Meta-Analysis Rationale

For systematic reviews / meta-analyses, authors need to provide the following information:

1.The rationale for conducting the systematic review / meta-analysis.

**Reply:**

The rationale for conducting this systematic review and meta-analysis is driven by the need to improve prognostic tools for endometrial cancer (EC), as traditional factors such as FIGO staging, lymph node metastasis, and lymph-vascular space invasion, though significant, are insufficient for accurately predicting recurrence, particularly in low-risk patients. This study aims to evaluate the lymphocyte-to-monocyte ratio (LMR) as a novel biomarker that could enhance risk estimation of metastasis at diagnosis and future recurrence, thus improving patient management and outcomes. LMR, obtainable through a routine preoperative blood examination, reflects the balance between lymphocytes and monocytes, which are indicators of immune response and inflammation. Previous research has shown the potential prognostic value of LMR in various cancers, including EC, but with inconsistent results. By consolidating existing research, this meta-analysis seeks to provide more compelling evidence regarding LMR's influence on EC prognosis and validate its robustness and consistency across different demographics such as race, age, and sample size. Furthermore, understanding the biological mechanisms linking inflammation and cancer supports LMR's role in prognosis, given the functions of lymphocytes and monocytes in tumor progression and immune response. Incorporating LMR into current prognostic models could enhance risk stratification and treatment planning for EC patients, especially for those at higher risk of recurrence despite traditional low-risk classifications. Thus, this systematic review and meta-analysis aim to provide a clearer and more comprehensive understanding of LMR's prognostic value in EC, potentially leading to improved patient management and personalized treatment approaches.

2.The contribution that it makes to knowledge in light of previously published related reports, including other meta-analyses and systematic reviews.

**Reply:**

The systematic review and meta-analysis contribute significantly to the existing body of knowledge by addressing the inconsistencies and gaps in previous research regarding the prognostic value of the lymphocyte-to-monocyte ratio (LMR) in endometrial cancer (EC). Prior studies have indicated the potential of LMR as a prognostic marker in various cancers, including EC, but have yielded inconsistent results. By synthesizing data from multiple studies, this meta-analysis provides a more robust and comprehensive evaluation of LMR's prognostic significance, offering clearer insights into its role in predicting overall survival (OS) and disease-free survival (DFS) in EC patients.

Moreover, this study adds to the knowledge by examining the prognostic value of LMR across diverse patient demographics, such as race, age, and sample size, thus ensuring the generalizability of the findings. It also integrates the biological mechanisms underlying the relationship between inflammation, immune response, and cancer progression, thereby elucidating the rationale behind LMR's prognostic capabilities.

In comparison to previous meta-analyses and systematic reviews, this study uniquely emphasizes the integration of LMR into current prognostic models for EC, potentially leading to enhanced risk stratification and personalized treatment planning. The findings underscore the importance of incorporating novel biomarkers like LMR into clinical practice to improve the accuracy of prognostic assessments and patient outcomes, particularly for those at higher risk of recurrence despite traditional low-risk classifications. This systematic review and meta-analysis thus fill a critical gap in the literature, offering evidence-based support for the clinical utility of LMR in managing EC and contributing to the advancement of personalized oncology.
